# Editorial: Challenges of failure learning and error management

**DOI:** 10.3389/fpsyg.2023.1286327

**Published:** 2023-10-25

**Authors:** Katrin Muehlfeld, Juergen Seifried, Thorsten Semrau, Jost Sieweke

**Affiliations:** ^1^Business Administration, University of Trier, Trier, Germany; ^2^Business School, University of Mannheim, Mannheim, Germany; ^3^School of Business and Economics, Vrije Universiteit Amsterdam, Amsterdam, Netherlands

**Keywords:** error management, error learning, failure learning, error management culture (EMC), error diagnosis, error beliefs

## The Research Topic articles

Errors and failures occur in both organizational and educational contexts, and they have the potential to promote learning and growth. This Research Topic is dedicated to improving our understanding of the opportunities associated with error management, the acquisition of knowledge from errors and failures, and strategies for harnessing this potential.

**Figure 1:**
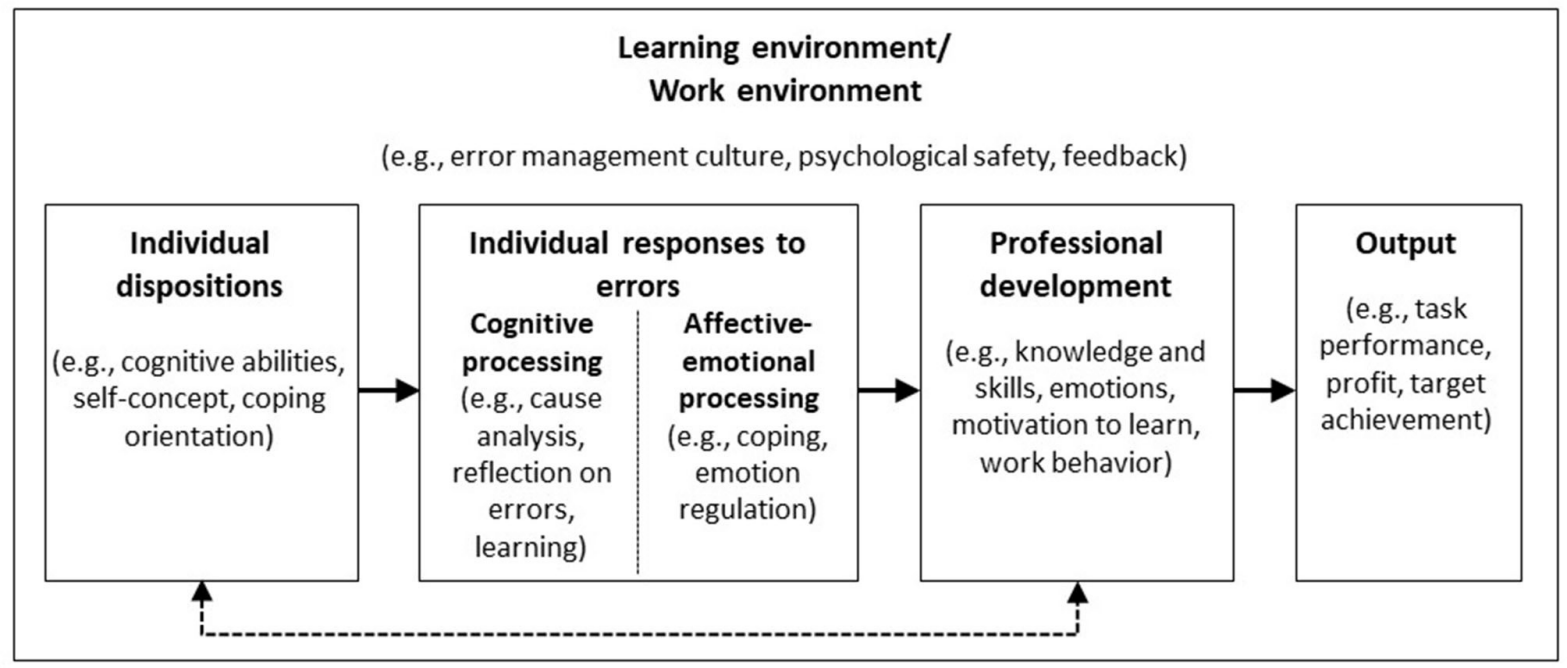
The error learning process.

The articles in this Research Topic represent different theoretical perspectives (organizational theory, educational theory), span different fields (high-risk organizations, higher education, teacher education), and use a range of methodological approaches (case studies, simulations, experiments). They address both individual dispositions and elements of learning and work environments that are conducive to learning, and they examine individual responses to errors as well as the outcomes that result from the error-learning process (Figure 1).

Dispositions are crucial in determining whether and to what extent individuals learn from errors and failures. In this Research Topic, the study contributed by Thiel and Semrau addresses how conscientiousness and extraversion help to explain individuals' responses to failure feedback. The findings highlight that learning from failure feedback for future task performance is more likely among individuals who are highly conscientious and/or highly extraverted. Complementing this research, the study by Hübner and Pfost explores students' beliefs about errors in higher education and their potential influence on learning behavior. The study demonstrates that such beliefs indeed affect students' readiness to participate in error-prone scenarios during seminar sessions. Interestingly, however, the authors also find that beliefs about errors do not appear to affect students' engagement in academic risks in front of their peers. In the field of teacher education, Hoth et al. observe that teachers' ability to deal with student errors is primarily influenced by their constructivist beliefs, which emphasize student-directed learning processes. Moreover, the ability to quickly identify common student errors is closely related to teachers' mathematical content knowledge.

This Research Topic also presents several manuscripts focusing on the influence of contextual factors residing within the work and learning environment. Specifically, there are three studies addressing error learning within organizations that examine the impact of error management culture, psychological safety, and perceptions of the error management climate. The study conducted by van Mourik et al. emphasizes the importance of perceptions of the error management climate in determining how error consequences affect learning. Elsayed et al. provide evidence to suggest that error risk-taking is critical in explaining why perceived psychological safety encourages employees to invest their time and effort in developing, introducing, and implementing innovative ideas in the workplace. In a study examining the errors made as an oil company adopted new technology to access untapped reserves, Van der Byl and Vrendenburg find that, while an error management culture (EMC) was prevalent in the company, error prevention measures were lacking.

When considering approaches to designing learning environments that stimulate error-based learning process, the work of Wesenberg et al. makes an important contribution. The authors analyze how examples of both correct and incorrect work can improve students' learning performance. They observe that the order in which correct and incorrect worked examples are introduced affects cognitive load and learning performance. Specifically, they find that presenting correct examples followed by incorrect ones results in lower cognitive load and better learning performance. Arenas et al. discuss how different ways of framing errors and negative feedback (error promotion vs. error prevention) affect performance and decision-making processes in complex tasks. They find that an error-prevention frame tends to prolong decision-making and emphasize the importance of the fit between task-specific demands (e.g., flexibility vs. thorough analysis) and the design of the learning environment, which may encourage either error promotion or error prevention.

## Future research directions

Based on the studies presented in this Research Topic, we would like to outline some challenges for future research in the field of error learning.

- *Deepening the understanding of error-learning processes* is necessary to gain a more comprehensive understanding of how individuals, teams, and organizations learn from errors. This includes examining in more detail the mechanisms involved in different forms of learning, such as experiential or vicarious learning.
- *Overcoming the limitations of self-reported data:* self-reports can be affected by bias and may not always accurately capture error learning. Future research could focus on developing more objective and reliable methods to collect error-learning data (e.g., wearable devices, sensors, or digital platforms to track behaviors and responses in real time).
- *Obtaining data from multiple sources* can provide a more holistic view of the processes involved. Approaches to integrate data from multiple channels, such as performance metrics, communication logs, or physiological measurements, appear to be beneficial.
- *Replication studies* are critical to validate the results of baseline research. This could include developing standardized protocols, ensuring consistent measurement procedures, and accounting for potential confounds.
- Error learning is a dynamic process that occurs over time. Conducting *longitudinal studies* would allow tracking of the ways in which error learning develops, changes, and impacts individuals, groups, and organizations over time.
- *Establishing clear output and outcome criteria* is essential in evaluating the effectiveness of error learning. There is an urgent need to identify relevant metrics at different levels, such as improvement in individual performance, improvement in team communication, and organizational adaptability.

We believe that addressing these challenges in future research can contribute to a deeper understanding of error-learning processes and their impact on individuals, teams, and organizations. As technologies and methods evolve, researchers will have more tools at their disposal to address these challenges and make important contributions to the field.

## Author contributions

KM: Conceptualization, Writing—original draft. JSe: Conceptualization, Writing—original draft. TS: Conceptualization, Writing—original draft. JSi: Writing—original draft, Conceptualization.

